# NO Is Not the Same as GSNO in the Regulation of Fe Deficiency Responses by Dicot Plants

**DOI:** 10.3390/ijms241612617

**Published:** 2023-08-09

**Authors:** Francisco Javier Romera, María José García, Carlos Lucena, Macarena Angulo, Rafael Pérez-Vicente

**Affiliations:** 1 Department of Agronomy (DAUCO María de Maeztu Unit of Excellence 2021–2023), Campus de Excelencia Internacional Agroalimentario, Universidad de Córdoba, 14071 Córdoba, Spain; ag1roruf@uco.es (F.J.R.); b02anpem@uco.es (M.A.); 2 Department of Botany, Ecology and Plant Physiology, Campus de Excelencia Internacional Agroalimentario, Universidad de Córdoba, 14071 Córdoba, Spain; b42lulec@uco.es (C.L.); bv1pevir@uco.es (R.P.-V.)

**Keywords:** dicotyledonous, ethylene, glutathione, GSNO reductase, iron, nitric oxide, *S*-nitrosoglutathione, Fe deficiency responses

## Abstract

Iron (Fe) is abundant in soils but with a poor availability for plants, especially in calcareous soils. To favor its acquisition, plants develop morphological and physiological responses, mainly in their roots, known as Fe deficiency responses. In dicot plants, the regulation of these responses is not totally known, but some hormones and signaling molecules, such as auxin, ethylene, glutathione (GSH), nitric oxide (NO) and *S*-nitrosoglutathione (GSNO), have been involved in their activation. Most of these substances, including auxin, ethylene, GSH and NO, increase their production in Fe-deficient roots while GSNO, derived from GSH and NO, decreases its content. This paradoxical result could be explained with the increased expression and activity in Fe-deficient roots of the GSNO reductase (GSNOR) enzyme, which decomposes GSNO to oxidized glutathione (GSSG) and NH_3_. The fact that NO content increases while GSNO decreases in Fe-deficient roots suggests that NO and GSNO do not play the same role in the regulation of Fe deficiency responses. This review is an update of the results supporting a role for NO, GSNO and GSNOR in the regulation of Fe deficiency responses. The possible roles of NO and GSNO are discussed by taking into account their mode of action through post-translational modifications, such as *S*-nitrosylation, and through their interactions with the hormones auxin and ethylene, directly related to the activation of morphological and physiological responses to Fe deficiency in dicot plants.

## 1. Introduction

Iron (Fe) is very abundant in most soils, mainly as Fe^3+^, although its availability to plants is low, especially in calcareous soils [1,2]. This low availability is mainly related to the low solubility of Fe oxides and hydroxides at a high pH [2]. Dicot (Strategy I) plants, such as Arabidopsis and the tomato, need to reduce Fe^3+^, abundant in most soils, to Fe^2+^, by means of a ferric reductase (encoded by *FRO2* in Arabidopsis) at the root surface, prior to its subsequent uptake through an Fe^2+^ transporter (encoded by *IRT1* in Arabidopsis; Refs. [3,4]). When grown under an Fe deficiency, dicot plants develop several physiological and morphological responses, mainly in roots, known as Fe deficiency responses and aimed at facilitating Fe mobilization and uptake (see Section 4; Refs. [3,5]). Once Fe has been acquired in enough quantity, Fe deficiency responses need to be switched off, to save energy and to avoid toxicity. It should be noted that the responses spend a lot of energy and resources for being activated and that Fe in excess can be very toxic. Among other effects, Fe can cause the formation of extremely reactive hydroxyl radicals through the Fenton reaction [4,6]. To solve both problems, a low availability of Fe in soils and toxicity when Fe is acquired in excess, plants have evolved sophisticated mechanisms to tightly control Fe acquisition and homeostasis [4]. In dicot plants, several hormones and signaling molecules, including ethylene, auxin, nitric oxide (NO) and glutathione (GSH), have been implicated in the activation of most physiological and morphological responses to Fe deficiency [3,5,7,8,9,10,11,12,13]. All the above cited substances enhance their production in Fe-deficient roots and are closely inter-related in a complex manner since some of them can affect the production and/or distribution of the other ones (see Section 6; Refs. [8,9,14]). Paradoxically, *S*-nitrosoglutathione (GSNO), which is formed from GSH and NO, decreases its production in Fe-deficient roots, where it has also been involved in the regulation of Fe deficiency responses [11,15,16]. These results suggest that GSNO, which is considered a relatively stable store for NO and a vehicle for its long-distance transport [17], does not play exactly the same roles that NO does in the regulation of Fe-deficient responses.

## 2. NO and GSNO in Plants

NO is a simple gaseous molecule (NO) which, due to its free radical nature, can react with cellular targets to form reactive nitrogen species, such as *S*-nitrosothiols, including *S*-nitrosoglutathione (GSNO; see below; Ref. [18]). NO can also bind to transition metal ions, such as Fe or Cu, to form metal–nitrosyl complexes, and to fatty acids to form nitro-fatty acids, which play an important role in the adaptation of plants to abiotic stresses [19,20]. NO is lipophilic and can easily diffuse across membranes [21]. In the 1980s, NO was recognized as a signaling molecule in mammals and later on, around the 1990s, also in plants [19,21]. NO has been involved in a myriad of developmental and physiological processes of plants, including seed germination, stomatal closure, flower development, root branching and senescence [20,22,23,24,25]. In addition, NO has been implicated in the responses of plants to both biotic and abiotic stresses, including pathogen infection, salinity, drought stress, nutrient deficiencies, an excess of heavy metals and others (see Section 3; Refs. [20,22,23,26,27,28,29,30,31,32]). 

NO synthesis has been reported in different plant organs, including roots, stems, leaves and flowers, and, at the subcellular level, both in the apoplast and in the symplast, and in different organelles, such as mitochondria, chloroplasts and peroxisomes [20,33]. NO can be synthesized by both enzymatic and non-enzymatic pathways [19,20,22,24,33]. In mammals, NO synthases (NOS) catalyze the conversion of L-arginine to NO and citrulline. However, although there is some evidence of NOS-like activity in plants, until now, no functional NOS protein has been isolated and characterized in higher plants [20,22]. It seems that, in plants, nitrate reductase (NR) plays the main role in the enzymatic NO production [19,20]. There are mutants impaired in NO production, including the Arabidopsis *nia1nia2* mutants, altered in the *NIA1* and *NIA2* genes, encoding NRs [19,34], and mutants that overproduce NO, such as the Arabidopsis *cue*/*nox1* mutants [19,35,36]. NO is usually detected and determined by using the permeable NO-sensitive fluorophore 4-amino-5-methylamino-2′,7′-difluoro-fluorescein diacetate (DAF-FM DA) [13,34].

NO is considered a phytohormone for some authors [37] while other ones do not consider it as such since no specific receptors for NO have been identified [21]. The action of NO is mainly mediated with its roles through post-translational modifications, including tyrosine nitration, metal nitrosylation and *S*-nitrosylation [19,25]. Tyrosine nitration is mediated with the peroxynitrite anion (OONO^−^), formed from NO and a superoxide radical, while metal nitrosylation is due to the interaction of NO with metalloproteins [19,25]. *S*-nitrosylation is a redox-based covalent addition of NO to the sulfhydryl group of a cysteine on a target protein and is the most prominent of the post-translational modifications caused with NO [19,25]. It can affect protein activity (activation or inhibition), translocation and protein function [38]. *S*-nitrosylation is a reversible process, involving nitrosylation, denitrosylation and transnitrosylation. The nitrosylation is considered a non-enzymatic process mediated directly with NO, or indirectly with *S*-nitrosothiols (e.g., GSNO) or other NO-derived compounds [19,25]. The specificity of the protein *S*-nitrosylation is mainly determined with the structure of the target protein and the local NO concentration in its vicinity [19]. Denitrosylation is mediated by the thioredoxin system and the transnitrosylation, the transfer of a NO group from one protein to another, is mediated by transnitrosylases, not yet found in plants [19,25].

**Figure 1:**
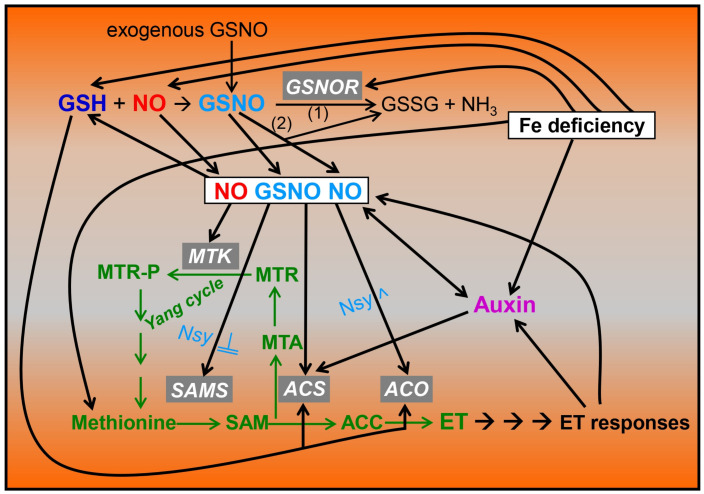
Inter-relationship between ethylene, auxin, GSH, NO and GSNO in Fe-deficient roots of dicot plants. Fe deficiency causes an increased production of ethylene (ET), auxin, glutathione (GSH) and nitric oxide (NO) in dicot roots, as well as an increased GSNOR expression and activity, which diminishes *S*-nitrosoglutathione (GSNO) content. GSNO can be enzymatically decomposed (1) to oxidized glutathione (GSSG) and NH_3_ by GSNOR or non-enzymatically (2) to generate GSSG and NO. NO (also the one originated from GSNO) and/or GSNO can influence ethylene synthesis by positively affecting the transcription of the *MTK*, *SAMS*, *ACS* and *ACO* genes; by positively affecting ACO activity with *S*-nitrosylation; or by negatively affecting SAMS activity with *S*-nitrosylation. GSH itself can also affect ethylene synthesis by affecting the transcription of *ACS* and *ACO* genes. Auxin can influence ethylene synthesis by affecting the transcription of *ACS* genes and NO synthesis. NO can affect GSH synthesis, and both NO and ethylene can affect auxin accumulation, distribution and signaling. Based on [8,9,11,13,14,15,16,35,38,40,53,54,55,56,57]. Nsy: Nitrosylation (^ or →: promotion; ─╢: inhibition). Green arrows and words indicate steps and compounds implicated in ethylene synthesis.

As previously stated, *S*-nitrosothiols are NO-derived compounds. Among them, GSNO is the most abundant low-molecular one and is formed non-enzymatically from glutathione (GSH) and NO under aerobic conditions (Figure 1; Refs. [17,18,32,39]. GSH and NO are inter-related since NO can influence GSH synthesis in roots [40]. Since the NO lifetime is relatively short (less than 10 s), GSNO is considered a relatively stable store for NO, being its main reservoir and a vehicle for its long-distance transport [17,18,39,41]. The levels of GSNO can be determined with several methods, including LC-ES/MS, chemiluminescence-based methods and a diamino-rhodamine fluorimetric-based method [15,42]. GSNO is tightly controlled with the GSNO reductase (GSNOR) enzyme, which decomposes it to oxidized glutathione (GSSG) and NH_3_ (Figure 1; Refs. [41,43,44]). GSNOR is a class III Alcohol Dehydrogenase, which was originally identified as a GSH-dependent Formaldehyde Dehydrogenase [18,43,45,46,47,48]. *GSNOR* is expressed in both roots and shoots [49]. In Arabidopsis, *GSNOR1* is the only gene encoding this enzyme [50]. There are Arabidopsis mutants that present a loss of *AtGSNOR1* function, such as the *gsnor1-3* mutant, which has higher GSNO contents, and Arabidopsis mutants that overexpress *AtGSNOR1*, such as the *gsnor1-1* mutant, which has lower GSNO contents [19,36,50]. Besides mutants, there are also transgenic lines that overexpress *GSNOR*, and transgenic lines where *GSNOR* is blocked by RNAi [51,52,53]. In addition to its decomposition with GSNOR, GSNO can be non-enzymatically decomposed to generate NO and GSSG in the presence of reductants (including GSH and ascorbate) and Cu (Figure 1; Refs. [17,18,32]). 

Similarly to NO, GSNO and, consequently, GSNOR have also been involved in the responses of plants to different biotic and abiotic stresses [27,39,43,44,45,48,50,52]. GSNOR activity generally increases under stress conditions [45,48]. Recently, Rudolf et al. [23] proposed that GSNO promotes the methylation of the repressive chromatin mark H3K9, which would impair the expression of stress-responsive genes. According to this proposal, GSNOR, by degrading GSNO, would positively affect the expression of stress-responsive genes [23].

## 3. Role of NO and GSNO in the Responses of Plants to Mineral Stresses

A mineral stress is related to the sub-optimal availability of essential elements or toxicity of essential and non-essential elements, including Al, Na, Cl and others [58]. As previously stated, both NO and GSNO/GSNOR participate in the responses of plants to many abiotic stresses, including mineral stresses, salinity, drought and others (see Section 2; Refs. [26,46,48]). In relation to mineral stresses, NO and/or GSNO have been implicated in several of them, including responses to heavy metals (e.g., Cu, Fe, Cd or Al; Refs. [27,32,59,60,61]), to metalloids [62], to salinity [37,63,64] or to nutrient deficiencies (see below). In general, NO production increases in roots under several mineral stresses. In addition, and similarly to what occurs with Fe deficiency (see Section 5), this increased NO production can be accompanied by an increased GSNOR activity and a decreased GSNO content. For example, Leterrier et al. [65] found increased NO and GSNOR activity in arsenic-treated *Arabidopsis* plants while GSNO decreased. 

NO and GSNO/GSNOR have been found to play key roles in the regulation of Fe deficiency responses by dicot (Strategy I) plants (see Section 5) but also in responses and adaptations to other nutrient deficiencies [56,66,67,68,69,70]. NO has been involved in the regulation of responses to P deficiency, including the development of cluster roots and root hairs, and the upregulation of phosphate transporters [66,69]. In relation to S deficiency, NO improves the adaption of plants to the oxidative stress caused by this deficiency, probably by maintaining the redox state through the ascorbate-GSH cycle [70]. Under a Mg deficiency, as occurs with Fe and P deficiency, NO has been implicated in the development of subapical root hairs [56,67]. In the studies about the role of NO in the regulation of P, S or Mg deficiency responses, exogenous GSNO has been used as a NO donor but what rarely has been studied is the role of endogenous GSNO and GSNOR in the regulation of these responses [71]. Nonetheless, *GSNOR1* expression also increases in P- and S-deficient roots, as occurring under an Fe deficiency (see Section 5; Ref. [15]). 

## 4. Fe Deficiency Responses in Dicot Plants

**Figure 2:**
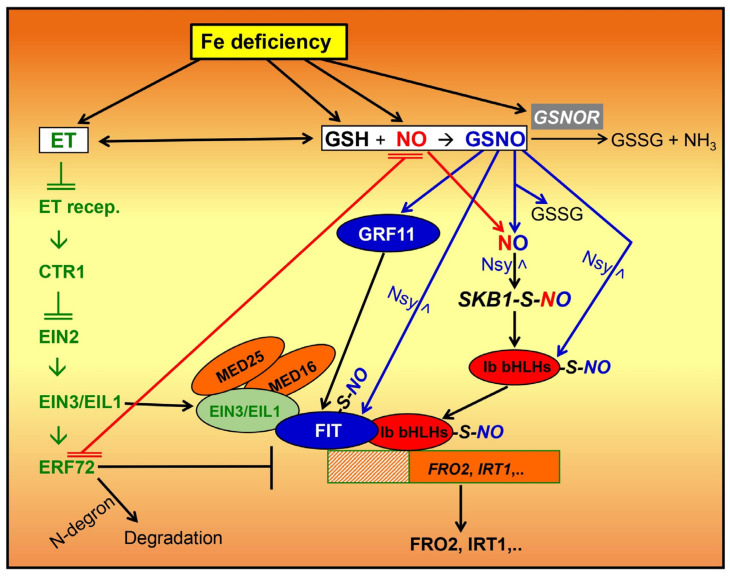
Working model proposed to explain the role of ethylene, NO and GSNO in the regulation of Fe acquisition genes by Arabidopsis. Fe deficiency causes an increased production of ethylene (ET), glutathione (GSH) and nitric oxide (NO), and a decreased GSNO content (see Figure 1). Ethylene activates the transcription of the Fe acquisition genes *FRO2* and *IRT1* by affecting FIT transcription and activity through the EIN3/EIL1 TFs, which interact with the MEDIATOR TFs MED16 and MED25. FIT transcription could also be affected by GSNO through the GRF11 TF, and FIT stability with GSNO, perhaps through *S*-nitrosylation. NO/GSNO could also activate the transcription of the *Ib bHLH* genes, by affecting the *S*-nitrosylation of the SKB1 protein. When denitrosylated, SKB1 is an epigenetic negative modulator that controls the expression of the *Ib bHLH* genes. GSNO could also affect the activity of the Ib bHLH TFs with their direct *S*-nitrosylation. FIT, along with Ib bHLH TFs, would activate *FRO2* and *IRT1* expression. ERF72, implicated in the inhibition of the expression of Fe acquisition genes, could be degraded by the N-degron (previously, N-end rule) pathway with the participation of NO. On the other hand, ERF72 can inhibit NO production. Based on [3,7,12,13,15,16,34,75,76,77,78,79,80,81]. (^ or →: promotion; ─╢: inhibition). Red lines indicate the processes probably affected by NO and blue lines indicate the ones probably affected by GSNO. NO (NblueOblue) indicates NO originated from GSNO while NO’ (NredOblue) indicates NO originated from either NO or GSNO. Green lines and words indicate steps and components implicated in ethylene signaling.

When grown under an Fe deficiency, dicot (Strategy I) plants develop several physiological and morphological responses, mainly in roots, known as Fe deficiency responses and aimed at facilitating Fe mobilization and uptake [3,4,5,72]. Among the physiological responses are an enhanced ferric reductase activity (due to a higher expression of the *FRO* gene); an enhanced Fe^2+^ uptake capacity (due to a higher expression of the *IRT1* gene); the acidification of the rhizosphere (due to a higher expression of *HA* (H^+^-ATPase) genes); and an increase in the synthesis and release of organic acids (e.g., citrate and malate), phenols (e.g., coumarins) and flavins to the medium [2,3,4,5,72,73,74]. The acidification facilitates the solubilization of Fe hydroxides and the functioning of the ferric reductase, which has an optimum pH around 5.0 [73]. Organic acids, phenols and flavins can act as chelating and/or reducing agents for Fe in the soil or inside the plant [2,3,74]. Among the morphological responses are the development of subapical root hairs, of cluster roots and of transfer cells, all of them aimed at increasing the surface of contact with the soil [3,9]. Both physiological and morphological responses are located in the subapical regions of the roots [3]. The activation of the Fe deficiency responses is not fully understood, but in recent years, several transcription factors (TFs) that participate in the upregulation of most of their associated genes have been found [3,4,5,6,72]. In Arabidopsis, the master regulator of most of these genes is FIT (bHLH29), a homolog of the tomato FER gene (Figure 2; Refs. [3,6]). The FIT regulatory network comprises other bHLH TFs of the Ib subgroup, including bHLH38, bHLH39, bHLH100 and bHLH101 (Figure 2). All of them have redundant functions and can interact with FIT to form heterodimers that activate the expression of the Fe acquisition genes *FRO2* and *IRT1* [4,5,72]. *FIT* is exclusively induced in roots in response to Fe deficiency while the other *Ib bHLH* genes cited above are induced in both roots and leaves in response to Fe deficiency [5]. 

The expression of *FIT* and *Ib bHLHs* is induced with homo- and hetero-dimers formed with IVc-subgroup bHLH TFs: bHLH34, bHLH104, bHLH105 (ILR3) and bHLH115 [4,72]. Upstream of the IVc subgroup, there are other bHLH TFs, such as bHLH121 (URI), and the BRUTUS (BTS) and BTS-LIKE (BTSL) proteins [4,72]. BTS and BTSL proteins are E3 ligases, which act as potential Fe sensors that interact with IVc bHLH TFs and FIT, targeting them for proteasomal degradation. Since FIT and the IVc bHLH TFs act as positive regulators of Fe deficiency responses, BTS and BTSL proteins act as negative regulators [4,72,82,83,84]. In recent years, it has been found that some peptides or small proteins, called IMAs (Iron Man/Fe-Uptake-Inducing Peptide: IMA/FEP), play a key role in the activation of Fe deficiency responses [83,84,85,86,87]. IMAs could impair the interaction of BTS and BTSL proteins with FIT and IVc bHLH TFs, thus diminishing their proteasomal degradation and, consequently, favoring the activation of Fe deficiency responses in Arabidopsis roots [83,84].

## 5. Role of NO and GSNO in the Regulation of Fe Deficiency Responses by Dicot Plants

The influence of hormones and signaling molecules in the regulation of morphological and physiological responses to Fe deficiency by dicot plants has been studied since the beginning of the 1980s, with the pioneering works of Landsberg, Römheld and Marschner suggesting a role for auxin in such a process (reviewed in Romera et al. [9]). After that, Romera and Alcántara [88], based on the use of ethylene inhibitors and precursors, proposed a similar role for the plant hormone ethylene, another simple gaseous molecule (C_2_H_4_), in such a regulation. The ethylene hypothesis has been further confirmed with different experimental results, including the higher ethylene production of Fe-deficient roots; the higher expression of both ethylene synthesis and signaling genes in Fe-deficient roots; the role of ethylene in the expression of many key Fe-related genes, including *FIT*(*FER*), *FRO* and *IRT1*; and the alteration of Fe deficiency responses in ethylene defective mutants (see Section 6; Refs. [3,7,9,89,90,91]). Ethylene has also been implicated in the regulation of the responses to other nutrient deficiencies [92,93,94,95]. 

Several years after the implication of ethylene in the regulation of Fe deficiency responses, it was found that NO played a similar role in such a regulation (see Section 6; Ref. [34]). These authors showed that NO production was enhanced in Fe-deficient tomato roots; that the NO-scavenger 2-(4-carboxyphenyl)-4,4,5,5-tetramethylimidazoline-1-oxyl-3-oxide (cPTIO) blocked the enhanced ferric reductase activity and the expression of several key Fe acquisition genes, including *FER*, *FRO1* and *IRT1*, in Fe-deficient tomato roots; that the NO donor GSNO induced the development of subapical root hairs and the expression of several Fe acquisition genes in roots of tomato plants grown under low Fe conditions; and that NO-deficient mutants, such as the NR-deficient tomato *nia* mutants, were impaired in the activation of Fe deficiency responses [34]. After that, similar results have been found by other authors in different dicot plant species by determining NO in roots; by using NO scavengers (e.g., cPTIO), NR inhibitors (e.g., tungstate) or NOS inhibitors (e.g., L-NAME); by using NO donors (e.g., sodium nitroprusside, diethylamine-NONOate or GSNO); and by using NO-defective mutants [7,10,11,13,15,16,20,24,66,71,76,96,97,98,99,100,101,102]. It should be noted that sodium nitroprusside is not an adequate NO donor for Fe studies since it contains an Fe atom [20]. 

Besides the above results, it has also been found that NO participates in other processes related to Fe deficiency responses and/or to Fe acquisition. In relation to Fe deficiency responses, NO has also been implicated in the stabilization of the FIT protein, probably because, in its presence, FIT protein is less likely to be a target of proteasomal degradation [76]. Additionally, it has been found that the 14-3-3 protein GRF11 acts downstream of NO to upregulate *FIT* expression, which can activate the expression of Fe acquisition genes (Figure 2; Refs. [77,103]). In relation to other processes related to Fe acquisition, NO has been implicated in Fe immobilization in the root apoplast by decreasing the pectin methylation of the cell wall [104,105]. 

In addition to NO, GSNO has also been involved in the regulation of Fe deficiency responses similarly to what occurs with GSH [10,11,12,13,15,16]. Some authors have proposed that GSNO specifically mediates the Fe deficiency signal through FIT (see Section 7; Ref. [16]). Very recently, Shee et al. [12] found that FIT and some Ib bHLH TFs, including bHLH38 and bHLH101, can be *S*-nitrosylated, probably by GSNO, which would improve their stability (Figure 2). The expression of the *Ib bHLH* genes can be negatively affected by the Shk1 binding protein 1 (SKB1/PRMT5), because of its positive effect on the chromatin package [3,106]. Since SKB1 can be nitrosylated [107], it is possible that NO/GSNO, through the *S*-nitrosylation of SKB1, could activate the expression of *Ib bHLH* genes (Figure 2; Refs. [3,106]). In supporting this view, several *Ib bHLH* genes, including *bHLH38*, *bHLH39* and *bHLH100*, are upregulated in leaves of the Arabidopsis *gsnor1-3* mutant, which has a higher GSNO content [44]. 

Both NO and GSH production, in the same way that ethylene does, increase in Fe-deficient roots (Table 1; Refs. [9,11,15,34,66,101,108]). However, GSNO content, in contrast to its precursors GSH and NO, decreases in Fe-deficient Arabidopsis WT roots (Table 1; Refs. [11,15,16]). Moreover, GSNO levels in Fe-sufficient Arabidopsis *opt3-2* mutant roots and pea *dgl* mutant roots, which present the constitutive activation of Fe deficiency responses, are also lower than those in their respective Fe-sufficient WT roots [15]. So, it seems that increased NO but decreased GSNO is a prerequisite for the induction of Fe deficiency responses. These paradoxical results could be explained with the increased GSNOR activity in Fe-deficient roots of dicot plant species, such as Arabidopsis and the tomato [15,53], since GSNOR decomposes GSNO (see above). In accordance with these results, Fe-sufficient Arabidopsis *opt3-2* mutant roots (see above) present higher *GSNOR1* expression and activity than the Fe-sufficient WT ones [15]. In agreement with all these results, Wen et al. [53] found that *GSNOR* overexpression in tomato plants causes an enhancement of the ferric reductase activity and the upregulation of the Fe acquisition genes *FRO1* and *IRT1*. 

The role of NO in the regulation of Fe deficiency responses by dicot plants is also supported with other experimental results, besides the ones previously described. It has been found that beneficial rhizosphere microorganisms eliciting induced systemic resistance (ISR) can also induce Fe deficiency responses. The root-specific MYB72 TF, involved in both processes (ISR and Fe deficiency responses), and NO are required for the activation of both kinds of responses [115,116,117,118].

In addition to their role under Fe-deficient conditions, GSH and NO, similarly to ethylene, also play a role in the responses of plants to Fe excess. All these substances, ethylene, GSH and NO, increase their production upon Fe excess too (Table 1; Refs. [109,110,111,112]). In the case of GSNO, although it has not been determined under Fe excess conditions, it is known that GSNOR is required for root tolerance to Fe toxicity, probably by preventing cell death via inhibiting Fe-dependent nitrosative and oxidative cytotoxicity [60].

## 6. Interactions of NO and GSNO with Ethylene and Auxin in the Regulation of Fe Deficiency Responses by Dicot Plants

NO can interact with many hormones, such as strigolactones, salicylic acid, abscisic acid, auxin and ethylene, and signaling molecules, such as polyamines, reactive oxygen species and hydrogen sulfide, to exert its functions in the regulation of responses to biotic and abiotic stresses [18,29,33,55,71,119,120,121,122,123,124]. In the regulation of Fe deficiency responses by dicot plants, some of these interactions have already been described [8,9,24,71,105]. Nonetheless, to simplify the description of all the possible interactions, in this review, we will only describe the interactions of NO with ethylene and auxin. NO, ethylene and auxin have all been implicated in the activation of both morphological and physiological responses to Fe deficiency in dicot plants, and all of them activate the expression of similar key Fe acquisition genes [3,7,8,9,13,15,71,93,96,125]. In the case of NO and ethylene, all the Fe-related genes upregulated by NO in Fe-deficient roots of Arabidopsis and cucumber plants are similarly upregulated by ethylene [7,13]. Moreover, auxin, ethylene and NO have been described as the downstream signals more closely related to the direct activation of the morphological and physiological responses. As examples, the EIN3/EIL1 TFs, related to ethylene, directly interact with the master regulator FIT; the GRF11 protein, regulated with NO, affects *FIT* transcription; and auxin acts downstream of ethylene and NO in the development of subapical root hairs (Figure 2; Refs. [67,75,77,126]). Since hormones and signaling molecules are inter-related between them, it is possible that some other ones involved in the regulation of Fe deficiency responses could finally act through ethylene, auxin or NO [9,93,105]. 

As previously stated, ethylene participates in the activation of Fe deficiency responses by dicot plants (see Section 5). Ethylene is synthetized from the methionine amino acid, through a pathway including SAMS (SAM Synthetase), ACS (ACC Synthase) and ACO (ACC Oxidase) (Figure 1; Ref. [127]):

SAMS ACS ACO

Methionine → SAM → ACC → Ethylene (ET)

Its proposed mode of action (Figure 2; Refs. [80,128]) is

ET─╢ET receptors → CTR1─╢EIN2 → EIN3/EILs → ERFs → ET responses

In this pathway, CTR1 is a kinase, EIN2 is a protein located in the endoplasmic reticulum membrane and EIN3, EILs and ERFs are TFs [80,128]. Mutants in the CTR1 protein present constitutive responses to ethylene while those mutated in EIN2 or EIN3 are insensitive to ethylene [80,128]. Very recently, our group [91] found that Arabidopsis *ein2* mutants are impaired in the upregulation of the Fe acquisition genes *FRO2* and *IRT1*. This clearly suggests that EIN2, and consequently ethylene, is implicated in the activation of Fe acquisition genes. Furthermore, it has been shown that EIN3 and EIL1, two TFs in the ethylene signaling pathway (see above), are implicated in the transcription and activity of the master regulator FIT (Figure 2; Refs. [75,126]. 

Since NO plays a similar role to ethylene in the regulation of Fe deficiency responses (see Section 5; Ref. [34]), the question arose as to whether NO acts downstream of ethylene, or ethylene acts downstream of NO, or if both act in conjunction. Results from our group and others have shown that ethylene, NO, GSH and GSNO are inter-related since each can influence the production of the others [7,8,13,14,56]. NO, GSNO or GSH applied to Fe-sufficient Arabidopsis plants greatly induced the expression in roots of many genes involved in ethylene synthesis, including *SAMS*, *ACS*, *ACO* and 5-methilthioribose kinase (*MTK*) (see Figure 1; Refs. [13,14]). The ethylene synthesis genes induced with NO are also induced under an Fe deficiency, which suggests that this deficiency probably upregulates them through NO. NO/GSNO could also affect ethylene synthesis with the *S*-nitrosylation of ethylene synthesis enzymes (Figure 1). The Arabidopsis *SAMS* isoform, *SAMS1*, but not *SAMS2* and *SAMS3*, can be reversibly inhibited with *S*-nitrosylation, which would impair ethylene synthesis [15,38,55]. In contrast, the *S*-nitrosylation of an ACO enzyme in a tomato improves ethylene synthesis [57]. In the opposite direction, ACC (an ethylene precursor) applied to Fe-sufficient Arabidopsis and cucumber plants caused NO accumulation in the subapical region of their roots (Figure 1; Refs. [13,43]). Moreover, the application of ACC to the Arabidopsis ethylene-insensitive mutant *ein2-5* did not cause this NO accumulation [91]. Similarly, NO accumulation in Mg-deficient roots is impaired in this *ein2-5* mutant [56]. Ethylene probably increases NO content by activating enzymes involved in its synthesis, such as NR and NOS [56]. All these results suggest that ethylene influences NO accumulation and vice versa and agree with other nutrient-related root processes showing a mutual and generally synergistic effect between NO and ethylene [21,129]. This mutual influence could probably lead to the amplification of activating signals involved in the upregulation of Fe- and other nutrient-related genes. There are also some processes, including fruit ripening, de-etiolation and lateral root formation, that are regulated with NO/ethylene antagonism [21,64,130]. In the same way, ethylene can also negatively affect NO by increasing the NO scavenger Phytoglobin1 under hypoxia [131]. Besides ethylene synthesis, NO can also affect ethylene signaling. The ethylene response factor ERF72 (also named RAP2.3), which negatively regulates Fe deficiency responses in Arabidopsis [79], belongs to the group VII ERFs, which are sensors of NO and can be targeted for proteolysis degradation by the N-degron (previously named the N-end rule) pathway in the presence of NO [78]. Reciprocally, RAP2.3 can negatively control NO homeostasis and signaling (Figure 2; Ref. [81]).

In relation to GSNO, its interaction with ethylene is also feasible. As described above, the SAM Synthetase SAMS1, involved in ethylene synthesis, can be inhibited with *S*-nitrosylation [15,38,55]. In this way, higher GSNO levels (such as those found in Fe-sufficient roots) could contribute to the *S*-nitrosylation of the SAMS1 enzyme, and, consequently, to the inhibition of ethylene synthesis. In contrast, lower GSNO levels (such as those found in Fe-deficient WT roots and in Fe-sufficient *opt3-2* and *dgl* roots) could contribute to the denitrosylation of the SAMS1 enzyme and, consequently, to an increase in ethylene synthesis [15]. In supporting this view, silencing GSNOR in *Nicotiana attenuate*, which leads to a higher GSNO content, decreased the herbivore-induced accumulation of ethylene [132]. In the opposite direction, ethylene could decrease GSNO content since it has been shown that ACC (ethylene precursor) can induce *GSNOR1* expression [15]. The possible relationship between GSNO/GSNOR and ethylene is also feasible because both GSNOR [133] and several ethylene synthesis enzymes, such as MTK (Figure 1), induced in Fe-deficient roots [7,9] are located in the phloem [134]. In fact, GSNO is presumably phloem-mobile [39]. All the above results would imply that ethylene could simultaneously increase NO accumulation while decreasing GSNO content [15].

Similar to ethylene, auxin is also inter-related with NO. Several works have found that auxin can induce some Fe deficiency responses in plants by acting through NO [8,96,97,103,135,136]. In contrast, it has been shown that NO can affect auxin transport, accumulation and signaling [8,54,67,137]. Auxin can also interact with ethylene, since auxin can enhance ethylene synthesis by affecting ACS enzymes, while ethylene can affect auxin accumulation and distribution [8,67]. 

In conclusion, ethylene, auxin and NO/GSNO are closely inter-related in the regulation of Fe deficiency responses. In some cases, it seems that auxin acts upstream of NO/GSNO and ethylene, such as in the regulation of Fe acquisition genes (Figure 2), but in other ones, auxin probably acts downstream of NO/GSNO and ethylene, such as in the development of root hairs. 

## 7. Why Is NO Not the Same as GSNO in the Regulation of Fe Deficiency Responses?

Since NO can react with GSH to generate GSNO, and GSNO can be decomposed to generate NO (Figure 1), it is difficult to assign a specific role to either NO or GSNO in the regulation of Fe deficiency responses, or in other processes. For example, upon the application of exogenous GSNO, we cannot know whether it will act by itself or by generating NO (Figure 1). Moreover, since GSH, NO and GSNO can stimulate ethylene synthesis (Figure 1), it is also difficult to know whether the final executor of their different effects on Fe deficiency responses is themselves or ethylene. A first attempt to decipher the different roles of NO and GSNO in the regulation of Fe deficiency responses by dicot plants is the one made by Dr. Yeh’s group [16]. These authors, by using chemical screening, identified a small molecule, [3-amino-N-(3-methylphenyl)thieno [2,3-b]pyridine-2-carboxamide], named R7 (‘R’ denoting repressor) that downregulates *FIT* expression, and, consequently, *IRT1* and *FRO2* expression, but not the one of the *Ib bHLH* genes. R7 treatment did not affect cellular levels of NO or GSH but decreased the GSNO level in roots. Exogenously supplying GSNO, but not other NO donors, alleviated this inhibitory effect of R7 on *FIT* expression. After these results, the authors propose that GSNO specifically mediates the Fe deficiency signal to FIT while NO could mediate the signal to Ib bHLHs (Figure 2; Ref. [16]). Since *FIT* transcription can be activated by the GRF11 protein, which is upregulated with a GSNO treatment [77], it is possible that GSNO could mediate *FIT* transcription through the GRF11 protein while the one of *Ib bHLH* genes could be mediated with NO (Figure 2).

As stated above (see Section 5), it seems that increased NO but decreased GSNO in roots is a prerequisite for the induction of Fe deficiency responses. Nonetheless, it is also clear that GSNO content should not be too low, since R7, which inhibits GSNO accumulation, also blocks *FIT* transcription and the Fe deficiency responses depending on FIT [16]. In supporting this view, Guan et al. [51] found that *GSNOR* overexpression in Arabidopsis plants, which provokes a low GSNO content, causes a downregulation of the Fe acquisition gene *IRT1*. However, and as described in Section 5, Wen et al. [53] found that *GSNOR* overexpression in tomato plants causes an enhancement of the ferric reductase activity and the upregulation of the Fe acquisition genes *FRO1* and *IRT1*. So, in Arabidopsis, a lower GSNO content inhibits Fe deficiency responses while, in the tomato, it activates them. Perhaps, in Arabidopsis, *GSNOR* overexpression causes a too low GSNO content.

There are several possibilities to explain the differences between NO and GSNO. First, the NO lifetime is very short while GSNO is considered a relatively stable store for NO [17,18,39,41]. Second, the time course of NO and GSNO abundance after Fe starvation can be different: in fact, GSNOR is greatly upregulated in roots after very few hours of this deficiency [15]. Third, the NO and GSNO location can be different. Under an Fe deficiency, NO mainly accumulates in the subapical regions of the roots [13,118] while it is not yet known where the decrease in GSNO that occurs under this condition is located. At the cellular level, GSNOR is a nuclear and cytosolic enzyme while NO can also be synthesized in the apoplast or in organelles, such as chloroplasts [20,33,44,46,137]. Fourth, NO and GSNO do not always interact with the same protein thiols to cause *S*-nitrosylation [47,138]. Fifth, NO can also become covalently bound to transition metals (e.g., Fe), or to fatty acids (see Section 2; Refs. [19,20,36,39]). In fact, some authors consider that NO may have a wider range of biological activity relative to GSNO, and that both NO and GSNO can exhibit additive functions [36].

In the literature, there are results showing different effects of NO and GSNO on plant processes. As examples, NO causes salicylic acid accumulation while GSNO reduces its accumulation [39,52]; the phenotype of the Arabidopsis *gsnor1-3* mutant, which has higher GSNO contents, is different than the Arabidopsis *nox1* mutant, which overproduces NO [137].

## 8. Concluding Remarks and Future Perspectives

GSH, NO and GSNO have been involved in the activation of Fe deficiency responses by dicot plants. Both GSH and NO increase their production in Fe-deficient roots. However, GSNO, derived from GSH and NO, decreases its content in Fe-deficient roots. These paradoxical results could be explained with the increased expression and activity of the GSNOR enzyme, which decomposes GSNO, in Fe-deficient roots. The fact that NO content increases while GSNO decreases in Fe-deficient roots suggests that NO and GSNO do not play exactly the same role in the regulation of Fe deficiency responses. Since most of the effects of NO and GSNO in plant processes are related to post-translational modifications provoked with *S*-nitrosylation, it is tempting to speculate that perhaps both substances can modify different proteins. In this sense, some results, including those presented by Dr. Yeh’s group, suggest that GSNO could act through the master regulator FIT while NO could act through other TFs, including the Ib bHLH TFs. Nonetheless, more research is needed to decipher the intriguing relationship between NO and GSNO, and between them and other signals implicated in the regulation of Fe deficiency responses, such as GSH, ethylene and auxin.

## Figures and Tables

**Table 1:** Effects of Fe deficiency and Fe excess in the production of ethylene (ET), glutathione (GSH), nitric oxide (NO) and *S*-nitrosoglutatione (GSNO) by roots of different dicot plant species.

	Fe Sufficiency	Fe Deficiency	Fe Excess	Plant Species	References
ET	+	+ + + +	+ + + +	Pea Cucumber Squash Arabidopsis (*)	[9,89,108,109,110]
GSH	+	+ + + +	+ + + +	Sugar beet Arabidopsis Rice (**)	[11,15,111]
NO	+	+ + + +	+ + + +	Lupinus Tomato Arabidopsis Peanut	[15,66,101,104,112]
GSNO	+ + + +	+ +	n.d.	Arabidopsis Pea	[11,15,16]

(*) Ramírez et al. [113] did not find increased GSH in Fe-deficient Arabidopsis roots; (**) rice presents characteristics of Strategy I (dicot) and Strategy II (graminaceous) plants [114]; n.d.: not determined. +: an arbitrary quantity; + +: Increase in quantity; + + + +: a significative higher quantity.

## Data Availability

Not applicable.

## References

[1] Briat J.-F., Dubos C., Gaymard F. (2015). Iron Nutrition, Biomass Production, and Plant Product Quality. Trends Plant. Sci..

[2] Vélez-Bermúdez I.C., Schmidt W. (2023). Plant Strategies to Mine Iron from Alkaline Substrates. Plant. Soil..

[3] Lucena C., Romera F.J., García M.J., Alcántara E., Pérez-Vicente R. (2015). Ethylene Participates in the Regulation of Fe Deficiency Responses in Strategy I Plants and in Rice. Front. Plant Sci..

[4] Liang G. (2022). Iron Uptake, Signaling, and Sensing in Plants. Plant Commun..

[5] Brumbarova T., Bauer P., Ivanov R. (2015). Molecular Mechanisms Governing *Arabidopsis* Iron Uptake. Trends Plant Sci..

[6] Schwarz B., Bauer P. (2020). FIT, a Regulatory Hub for Iron Deficiency and Stress Signaling in Roots, and FIT-Dependent and -Independent Gene Signatures. J. Exp. Bot..

[7] García M.J., Lucena C., Romera F.J., Alcántara E., Pérez-Vicente R. (2010). Ethylene and Nitric Oxide Involvement in the Up-Regulation of Key Genes Related to Iron Acquisition and Homeostasis in Arabidopsis. J. Exp. Bot..

[8] Romera F.J., García M.J., Alcántara E., Pérez-Vicente R. (2011). Latest Findings about the Interplay of Auxin, Ethylene and Nitric Oxide in the Regulation of Fe Deficiency Responses by Strategy I Plants. Plant Signal. Behav..

[9] Romera F.J., Lucena C., García M.J., Alcántara E., Pérez-Vicente R. (2017). The Role of Ethylene and Other Signals in the Regulation of Fe Deficiency Responses by Dicot Plants. Stress Signaling in Plants: Genomics and Proteomics Perspective.

[10] Koen E., Szymańska K., Klinguer A., Dobrowolska G., Besson-Bard A., Wendehenne D. (2012). Nitric Oxide and Glutathione Impact the Expression of Iron Uptake- and Iron Transport-Related Genes as Well as the Content of Metals in *A Thaliana* Plants Grown under Iron Deficiency. Plant Signal. Behav..

[11] Shanmugam V., Wang Y.-W., Tsednee M., Karunakaran K., Yeh K.-C. (2015). Glutathione Plays an Essential Role in Nitric Oxide-Mediated Iron-Deficiency Signaling and Iron-Deficiency Tolerance in *Arabidopsis*. Plant J..

[12] Shee R., Ghosh S., Khan P., Sahid S., Roy C., Shee D., Paul S., Datta R. (2022). Glutathione Regulates Transcriptional Activation of Iron Transporters via S-Nitrosylation of BHLH Factors to Modulate Subcellular Iron Homoeostasis. Plant Cell Environ..

[13] García M.J., Suárez V., Romera F.J., Alcántara E., Pérez-Vicente R. (2011). A New Model Involving Ethylene, Nitric Oxide and Fe to Explain the Regulation of Fe-Acquisition Genes in Strategy I Plants. Plant Physiol. Biochem..

[14] Datta R., Kumar D., Sultana A., Hazra S., Bhattacharyya D., Chattopadhyay S. (2015). Glutathione Regulates ACC Synthase Transcription via WRKY33 and ACC Oxidase by Modulating MRNA Stability to Induce Ethylene Synthesis during Stress. Plant Physiol..

[15] García M.J., Corpas F.J., Lucena C., Alcántara E., Pérez-Vicente R., Zamarreño Á.M., Bacaicoa E., García-Mina J.M., Bauer P., Romera F.J. (2018). A Shoot Fe Signaling Pathway Requiring the OPT3 Transporter Controls GSNO Reductase and Ethylene in *Arabidopsis thaliana* Roots. Front. Plant Sci..

[16] Kailasam S., Wang Y., Lo J.-C., Chang H.-F., Yeh K.-C. (2018). S-Nitrosoglutathione Works Downstream of Nitric Oxide to Mediate Iron-Deficiency Signaling in Arabidopsis. Plant J..

[17] Corpas F.J., Leterrier M., Valderrama R., Airaki M., Chaki M., Palma J.M., Barroso J.B. (2011). Nitric Oxide Imbalance Provokes a Nitrosative Response in Plants under Abiotic Stress. Plant Sci..

[18] Lindermayr C. (2018). Crosstalk between Reactive Oxygen Species and Nitric Oxide in Plants: Key Role of S-Nitrosoglutathione Reductase. Free Radic. Biol. Med..

[19] Feng J., Chen L., Zuo J. (2019). Protein *S*-Nitrosylation in Plants: Current Progresses and Challenges. J. Integr. Plant Biol..

[20] Tewari R.K., Horemans N., Watanabe M. (2021). Evidence for a Role of Nitric Oxide in Iron Homeostasis in Plants. J. Exp. Bot..

[21] Kolbert Z., Feigl G., Freschi L., Poór P. (2019). Gasotransmitters in Action: Nitric Oxide-Ethylene Crosstalk during Plant Growth and Abiotic Stress Responses. Antioxidants.

[22] Domingos P., Prado A.M., Wong A., Gehring C., Feijo J.A. (2015). Nitric Oxide: A Multitasked Signaling Gas in Plants. Mol. Plant.

[23] Rudolf E.E., Hüther P., Forné I., Georgii E., Han Y., Hell R., Wirtz M., Imhof A., Becker C., Durner J. (2021). GSNOR Contributes to Demethylation and Expression of Transposable Elements and Stress-Responsive Genes. Antioxidants.

[24] Mahawar L., Ramasamy K.P., Pandey A., Prasad S.M. (2022). Iron Deficiency in Plants: An Update on Homeostasis and Its Regulation by Nitric Oxide and Phytohormones. Plant Growth Regul..

[25] Pande A., Mun B.G., Rahim W., Khan M., Lee D.S., Lee G.M., Al Azzawi T.N.I., Hussain A., Kim C.K., Yun B.W. (2022). Phytohormonal Regulation Through Protein S-Nitrosylation Under Stress. Front. Plant Sci..

[26] Fancy N.N., Bahlmann A., Loake G.J. (2017). Nitric Oxide Function in Plant Abiotic Stress. Plant Cell Environ..

[27] Sahay S., Gupta M. (2017). An Update on Nitric Oxide and Its Benign Role in Plant Responses under Metal Stress. Nitric Oxide.

[28] Nabi R.B.S., Tayade R., Hussain A., Kulkarni K.P., Imran Q.M., Mun B.G., Yun B.W. (2019). Nitric Oxide Regulates Plant Responses to Drought, Salinity, and Heavy Metal Stress. Environ. Exp. Bot..

[29] Zhou X., Joshi S., Khare T., Patil S., Shang J., Kumar V. (2021). Nitric Oxide, Crosstalk with Stress Regulators and Plant Abiotic Stress Tolerance. Plant Cell Rep..

[30] Khan M., Ali S., Al Azzawi T.N.I., Yun B.-W. (2023). Nitric Oxide Acts as a Key Signaling Molecule in Plant Development under Stressful Conditions. Int. J. Mol. Sci..

[31] Kumar D., Ohri P. (2023). Say “NO” to Plant Stresses: Unravelling the Role of Nitric Oxide under Abiotic and Biotic Stress. Nitric Oxide.

[32] Wang X., Du H., Ma M., Rennenberg H. (2023). The Dual Role of Nitric Oxide (NO) in Plant Responses to Cadmium Exposure. Sci. Total Environ..

[33] Singhal R.K., Jatav H.S., Aftab T., Pandey S., Mishra U.N., Chauhan J., Chand S., Indu, Saha D., Dadarwal B.K. (2021). Roles of Nitric Oxide in Conferring Multiple Abiotic Stress Tolerance in Plants and Crosstalk with Other Plant Growth Regulators. J. Plant Growth Regul..

[34] Graziano M., Lamattina L. (2007). Nitric Oxide Accumulation Is Required for Molecular and Physiological Responses to Iron Deficiency in Tomato Roots. Plant J..

[35] Fernández-Marcos M., Sanz L., Lewis D.R., Muday G.K., Lorenzo O. (2011). Nitric Oxide Causes Root Apical Meristem Defects and Growth Inhibition While Reducing PIN-FORMED 1 (PIN1)-Dependent Acropetal Auxin Transport. Proc. Natl. Acad. Sci. USA.

[36] Yun B., Skelly M.J., Yin M., Yu M., Mun B., Lee S., Hussain A., Spoel S.H., Loake G.J. (2016). Nitric Oxide and *S*-nitrosoglutathione Function Additively during Plant Immunity. New Phytol..

[37] Jahan B., Rasheed F., Sehar Z., Fatma M., Iqbal N., Masood A., Anjum N.A., Khan N.A. (2021). Coordinated Role of Nitric Oxide, Ethylene, Nitrogen, and Sulfur in Plant Salt Stress Tolerance. Stresses.

[38] Kovacs I., Ageeva A., König E.E., Lindermayr C. (2016). S-Nitrosylation of Nuclear Proteins: New Pathways in Regulation of Gene Expression. Adv. Bot. Res..

[39] Malik S.I., Hussain A., Yun B.-W., Spoel S.H., Loake G.J. (2011). GSNOR-Mediated de-Nitrosylation in the Plant Defence Response. Plant Sci..

[40] Innocenti G., Pucciariello C., Le Gleuher M., Hopkins J., De Stefano M., Delledonne M., Puppo A., Baudouin E., Frendo P. (2007). Glutathione Synthesis Is Regulated by Nitric Oxide in *Medicago truncatula* Roots. Planta.

[41] Corpas F.J., Alché J.D., Barroso J.B. (2013). Current Overview of S-Nitrosoglutathione (GSNO) in Higher Plants. Front. Plant Sci..

[42] Mioto P.T., Rodríguez-Ruiz M., Mot A.C., Zuccarelli R., Corpas F.J., Freschi L., Mercier H. (2017). Alternative Fluorimetric-Based Method to Detect and Compare Total S-Nitrosothiols in Plants. Nitric Oxide.

[43] Leterrier M., Chaki M., Airaki M., Valderrama R., Palma J.M., Barroso J.B., Corpas F.J. (2011). Function of S-Nitrosoglutathione Reductase (GSNOR) in Plant Development and under Biotic/Abiotic Stress. Plant Signal. Behav..

[44] Xu S., Guerra D., Lee U., Vierling E. (2013). S-Nitrosoglutathione Reductases Are Low-Copy Number, Cysteine-Rich Proteins in Plants That Control Multiple Developmental and Defense Responses in Arabidopsis. Front. Plant Sci..

[45] Kubienová L., Tichá T., Jahnová J., Luhová L., Mieslerová B., Petřivalský M. (2014). Effect of Abiotic Stress Stimuli on S-Nitrosoglutathione Reductase in Plants. Planta.

[46] Jahnová J., Luhová L., Petřivalský M. (2019). S-Nitrosoglutathione Reductase—The Master Regulator of Protein S-Nitrosation in Plant NO Signaling. Plants.

[47] Ventimiglia L., Mutus B. (2020). The Physiological Implications of S-Nitrosoglutathione Reductase (GSNOR) Activity Mediating NO Signalling in Plant Root Structures. Antioxidants.

[48] Li B., Sun C., Lin X., Busch W. (2021). The Emerging Role of GSNOR in Oxidative Stress Regulation. Trends Plant Sci..

[49] Kubienová L., Kopečný D., Tylichová M., Briozzo P., Skopalová J., Šebela M., Navrátil M., Tâche R., Luhová L., Barroso J.B. (2013). Structural and Functional Characterization of a Plant S-Nitrosoglutathione Reductase from *Solanum lycopersicum*. Biochimie.

[50] Kwon E., Feechan A., Yun B.W., Hwang B.H., Pallas J.A., Kang J.G., Loake G.J. (2012). AtGSNOR1 Function Is Required for Multiple Developmental Programs in Arabidopsis. Planta.

[51] Guan M.Y., Zhu Y.X., Liu X.X., Jin C.W. (2019). Induction of S-Nitrosoglutathione Reductase Reduces Root Cadmium Uptake by Inhibiting Iron-Regulated Transporter 1. Plant Soil..

[52] Hussain A., Yun B.W., Kim J.H., Gupta K.J., Hyung N.I., Loake G.J. (2019). Novel and Conserved Functions of S-Nitrosoglutathione Reductase in Tomato. J. Exp. Bot..

[53] Wen D., Sun S., Yang W., Zhang L., Liu S., Gong B., Shi Q. (2019). Overexpression of S-Nitrosoglutathione Reductase Alleviated Iron-Deficiency Stress by Regulating Iron Distribution and Redox Homeostasis. J. Plant Physiol..

[54] Terrile M.C., París R., Calderón-Villalobos L.I.A., Iglesias M.J., Lamattina L., Estelle M., Casalongué C.A. (2012). Nitric Oxide Influences Auxin Signaling through S-Nitrosylation of the Arabidopsis TRANSPORT INHIBITOR RESPONSE 1 Auxin Receptor. Plant J..

[55] Freschi L. (2013). Nitric Oxide and Phytohormone Interactions: Current Status and Perspectives. Front. Plant Sci..

[56] Liu M., Liu X.X., He X.L., Liu L.J., Wu H., Tang C.X., Zhang Y.S., Jin C.W. (2017). Ethylene and Nitric Oxide Interact to Regulate the Magnesium Deficiency-Induced Root Hair Development in Arabidopsis. New Phytol..

[57] Liu M., Wei J.W., Liu W., Gong B. (2023). S-Nitrosylation of ACO Homolog 4 Improves Ethylene Synthesis and Salt Tolerance in Tomato. New Phytol..

[58] Lynch J.P., St. Clair S.B. (2004). Mineral Stress: The Missing Link in Understanding How Global Climate Change Will Affect Plants in Real World Soils. Field Crops Res..

[59] Pető A., Lehotai N., Feigl G., Tugyi N., Ördög A., Gémes K., Tari I., Erdei L., Kolbert Z. (2013). Nitric Oxide Contributes to Copper Tolerance by Influencing ROS Metabolism in Arabidopsis. Plant Cell Rep..

[60] Li B., Sun L., Huang J., Göschl C., Shi W., Chory J., Busch W. (2019). GSNOR Provides Plant Tolerance to Iron Toxicity via Preventing Iron-Dependent Nitrosative and Oxidative Cytotoxicity. Nat. Commun..

[61] Pan C., Li X., Yao S., Luo S., Liu S., Wang A., Xiao D., Zhan J., He L. (2021). S-Nitrosated Proteomic Analysis Reveals the Regulatory Roles of Protein S-Nitrosation and S-Nitrosoglutathione Reductase during Al-Induced PCD in Peanut Root Tips. Plant Sci..

[62] Kolbert Z., Ördög A. (2021). Involvement of Nitric Oxide (NO) in Plant Responses to Metalloids. J. Hazard. Mater..

[63] Zhou S., Jia L., Chu H., Wu D., Peng X., Liu X., Zhang J., Zhao J., Chen K., Zhao L. (2016). Arabidopsis CaM1 and CaM4 Promote Nitric Oxide Production and Salt Resistance by Inhibiting S-Nitrosoglutathione Reductase via Direct Binding. PLoS Genet..

[64] Singh N., Bhatla S.C. (2018). Nitric Oxide Regulates Lateral Root Formation through Modulation of ACC Oxidase Activity in Sunflower Seedlings under Salt Stress. Plant Signal. Behav..

[65] Leterrier M., Airaki M., Palma J.M., Chaki M., Barroso J.B., Corpas F.J. (2012). Arsenic Triggers the Nitric Oxide (NO) and S-Nitrosoglutathione (GSNO) Metabolism in Arabidopsis. Environ. Pollut..

[66] Meng Z.B., Chen L.Q., Suo D., Li G.X., Tang C.X., Zheng S.J. (2012). Nitric Oxide Is the Shared Signalling Molecule in Phosphorus- and Iron-Deficiency-Induced Formation of Cluster Roots in White Lupin (*Lupinus albus*). Ann. Bot..

[67] Liu M., Zhang H., Fang X., Zhang Y., Jin C. (2018). Auxin Acts Downstream of Ethylene and Nitric Oxide to Regulate Magnesium Deficiency-Induced Root Hair Development in *Arabidopsis thaliana*. Plant Cell Physiol..

[68] Buet A., Galatro A., Ramos-Artuso F., Simontacchi M. (2019). Nitric Oxide and Plant Mineral Nutrition: Current Knowledge. J. Exp. Bot..

[69] Galatro A., Ramos-Artuso F., Luquet M., Buet A., Simontacchi M. (2020). An Update on Nitric Oxide Production and Role Under Phosphorus Scarcity in Plants. Front. Plant Sci..

[70] Siddiqui M.H., Alamri S., Alsubaie Q.D., Ali H.M., Khan M.N., Al-Ghamdi A., Ibrahim A.A., Alsadon A. (2020). Exogenous Nitric Oxide Alleviates Sulfur Deficiency-Induced Oxidative Damage in Tomato Seedlings. Nitric Oxide.

[71] García M.J., Lucena C., Romera F.J. (2021). Ethylene and Nitric Oxide Involvement in the Regulation of Fe and P Deficiency Responses in Dicotyledonous Plants. Int. J. Mol. Sci..

[72] Gao F., Dubos C. (2021). Transcriptional Integration of Plant Responses to Iron Availability. J. Exp. Bot..

[73] Waters B.M., Lucena C., Romera F.J., Jester G.G., Wynn A.N., Rojas C.L., Alcántara E., Pérez-Vicente R. (2007). Ethylene Involvement in the Regulation of the H^+^-ATPase CsHA1 Gene and of the New Isolated Ferric Reductase CsFRO1 and Iron Transporter CsIRT1 Genes in Cucumber Plants. Plant Physiol. Biochem..

[74] Tsai H.H., Schmidt W. (2017). Mobilization of Iron by Plant-Borne Coumarins. Trends Plant. Sci..

[75] Lingam S., Mohrbacher J., Brumbarova T., Potuschak T., Fink-Straube C., Blondet E., Genschik P., Bauer P. (2011). Interaction between the BHLH Transcription Factor FIT and Ethylene Insensitive3/Ethylene Insensitive3-LIKE1 Reveals Molecular Linkage between the Regulation of Iron Acquisition and Ethylene Signaling in Arabidopsis. Plant Cell.

[76] Meiser J., Lingam S., Bauer P. (2011). Posttranslational Regulation of the Iron Deficiency Basic Helix-Loop-Helix Transcription Factor FIT Is Affected by Iron and Nitric Oxide. Plant Physiol..

[77] Yang J.L., Chen W.W., Chen L.Q., Qin C., Jin C.W., Shi Y.Z., Zheng S.J. (2013). The 14-3-3 Protein GENERAL REGULATORY FACTOR11 (GRF11) Acts Downstream of Nitric Oxide to Regulate Iron Acquisition in *Arabidopsis thaliana*. New Phytol..

[78] Gibbs D.J., Conde J.V., Berckhan S., Prasad G., Mendiondo G.M., Holdsworth M.J. (2015). Group VII Ethylene Response Factors Coordinate Oxygen and Nitric Oxide Signal Transduction and Stress Responses in Plants. Plant Physiol..

[79] Liu W., Li Q., Wang Y., Wu T., Yang Y., Zhang X., Han Z., Xu X. (2017). Ethylene Response Factor AtERF72 Negatively Regulates *Arabidopsis thaliana* Response to Iron Deficiency. Biochem. Biophys. Res. Commun..

[80] Binder B.M. (2020). Ethylene Signaling in Plants. J. Biol. Chem..

[81] León J., Costa-Broseta Á., Castillo M.C. (2020). RAP2.3 Negatively Regulates Nitric Oxide Biosynthesis and Related Responses through a Rheostat-like Mechanism in Arabidopsis. J. Exp. Bot..

[82] Rodríguez-Celma J., Connorton J.M., Kruse I., Green R.T., Franceschetti M., Chen Y.T., Cui Y., Ling H.Q., Yeh K.C., Balk J. (2019). Arabidopsis BRUTUS-LIKE E3 Ligases Negatively Regulate Iron Uptake by Targeting Transcription Factor FIT for Recycling. Proc. Natl. Acad. Sci. USA.

[83] Li Y., Kai Lu C., Yang Li C., Hua Lei R., Na Pu M., Hui Zhao J., Peng F., Qian Ping H., Wang D., Liang G. (2021). IRON MAN Interacts with BRUTUS to Maintain Iron Homeostasis in Arabidopsis. Proc. Natl. Acad. Sci. USA.

[84] Lichtblau D.M., Schwarz B., Baby D., Endres C., Sieberg C., Bauer P. (2022). The Iron Deficiency-Regulated Small Protein Effector FEP3/IRON MAN1 Modulates Interaction of BRUTUS-LIKE1 with BHLH Subgroup IVc and POPEYE Transcription Factors. Front. Plant Sci..

[85] Grillet L., Lan P., Li W., Mokkapati G., Schmidt W. (2018). IRON MAN Is a Ubiquitous Family of Peptides That Control Iron Transport in Plants. Nat. Plants.

[86] Hirayama T., Lei G.J., Yamaji N., Nakagawa N., Ma J.F. (2018). The Putative Peptide Gene FEP1 Regulates Iron Deficiency Response in Arabidopsis. Plant Cell Physiol..

[87] García M.J., Angulo M., Romera F.J., Lucena C., Pérez-Vicente R. (2022). A Shoot Derived Long Distance Iron Signal May Act Upstream of the IMA Peptides in the Regulation of Fe Deficiency Responses in Arabidopsis thaliana Roots. Front. Plant Sci..

[88] Romera F.J., Alcantara E. (1994). Iron-Deficiency Stress Responses in Cucumber (*Cucumis Sativus* L.) Roots. A Possible Role for Ethylene?. Plant Physiol..

[89] Romera F.J., Alcántara E. (2004). Ethylene Involvement in the Regulation of Fe-Deficiency Stress Responses by Strategy I Plants. Funct. Plant Biol..

[90] Li W., Lan P. (2017). The Understanding of the Plant Iron Deficiency Responses in Strategy I Plants and the Role of Ethylene in This Process by Omic Approaches. Front. Plant Sci..

[91] Angulo M., García M.J., Alcántara E., Pérez-Vicente R., Romera F.J. (2021). Comparative Study of Several Fe Deficiency Responses in the *Arabidopsis thaliana* Ethylene Insensitive Mutants *Ein2-1* and *Ein2-5*. Plants.

[92] García M.J., Romera F.J., Lucena C., Alcántara E., Pérez-Vicente R. (2015). Ethylene and the Regulation of Physiological and Morphological Responses to Nutrient Deficiencies. Plant Physiol..

[93] Iqbal N., Gautam H., Khan M.I.R., Per T.S., Khan N.A., Umar S. (2023). Crosstalk between Ethylene and Mineral Nutrients in Regulation of Morphophysiological Traits and Nutrients Homeostasis in Plants. The Plant Hormone Ethylene.

[94] Ma B., Ma T., Xian W., Hu B., Chu C. (2023). Interplay between Ethylene and Nitrogen Nutrition: How Ethylene Orchestrates Nitrogen Responses in Plants. J. Integr. Plant Biol..

[95] Romera F.J., Smith A.P., Pérez-Vicente R. (2016). Editorial: Ethylene’s Role in Plant Mineral Nutrition. Front. Plant Sci..

[96] Chen W.W., Yang J.L., Qin C., Jin C.W., Mo J.H., Ye T., Zheng S.J. (2010). Nitric Oxide Acts Downstream of Auxin to Trigger Root Ferric-Chelate Reductase Activity in Response to Iron Deficiency in Arabidopsis. Plant Physiol..

[97] Jin C.W., Du S.T., Shamsi I.H., Luo B.F., Lin X.Y. (2011). NO Synthase-Generated NO Acts Downstream of Auxin in Regulating Fe-Deficiency-Induced Root Branching That Enhances Fe-Deficiency Tolerance in Tomato Plants. J. Exp. Bot..

[98] Kong J., Dong Y., Xu L., Liu S., Bai X. (2014). Role of Exogenous Nitric Oxide in Alleviating Iron Deficiency-Induced Peanut Chlorosis on Calcareous Soil. J. Plant Interact..

[99] Buet A., Simontacchi M. (2015). Nitric Oxide and Plant Iron Homeostasis. Ann. N. Y. Acad. Sci..

[100] Song Y.L., Dong Y.J., Tian X.Y., Wang W.W., He Z.L. (2017). Effects of Nitric Oxide and Fe Supply on Recovery of Fe Deficiency Induced Chlorosis in Peanut Plants. Biol. Plant.

[101] Song Y., Dong Y., Tian X., Wang W., He Z. (2018). Mechanisms of Exogenous Nitric Oxide and 24-Epibrassinolide Alleviating Chlorosis of Peanut Plants Under Iron Deficiency. Pedosphere.

[102] Kabir A.H., Ela E.J., Bagchi R., Rahman M.A., Peiter E., Lee K.-W. (2023). Nitric Oxide Acts as an Inducer of Strategy-I Responses to Increase Fe Availability and Mobilization in Fe-Starved Broccoli (*Brassica oleracea* Var. Oleracea). Plant Physiol. Biochem..

[103] Liu X.X., He X.L., Jin C.W. (2016). Roles of Chemical Signals in Regulation of the Adaptive Responses to Iron Deficiency. Plant Signal. Behav..

[104] Ye Y.Q., Jin C.W., Fan S.K., Mao Q.Q., Sun C.L., Yu Y., Lin X.Y. (2015). Elevation of NO Production Increases Fe Immobilization in the Fe-Deficiency Roots Apoplast by Decreasing Pectin Methylation of Cell Wall. Sci. Rep..

[105] Zhu X.F., Wang B., Song W.F., Zheng S.J., Shen R.F. (2015). Putrescine Alleviates Iron Deficiency via NO-Dependent Reutilization of Root Cell-Wall Fe in Arabidopsis. Plant Physiol..

[106] Fan H., Zhang Z., Wang N., Cui Y., Sun H., Liu Y., Wu H., Zheng S., Bao S., Ling H.-Q. (2014). SKB1/PRMT5-Mediated Histone H4R3 Dimethylation of Ib Subgroup BHLH Genes Negatively Regulates Iron Homeostasis in *Arabidopsis thaliana*. Plant J..

[107] Hu J., Huang X., Chen L., Sun X., Lu C., Zhang L., Wang Y., Zuo J. (2015). Site-Specific Nitrosoproteomic Identification of Endogenously S-Nitrosylated Proteins in Arabidopsis. Plant Physiol..

[108] Ye L., Li L., Wang L., Wang S., Li S., Du J., Zhang S., Shou H. (2015). MPK3/MPK6 Are Involved in Iron Deficiency-Induced Ethylene Production in Arabidopsis. Front. Plant Sci..

[109] Li G., Song H., Li B., Kronzucker H.J., Shi W. (2015). Auxin Resistant1 and PIN-FORMED2 Protect Lateral Root Formation in Arabidopsis under Iron Stress. Plant Physiol..

[110] Li G., Xu W., Kronzucker H.J., Shi W. (2015). Ethylene Is Critical to the Maintenance of Primary Root Growth and Fe Homeostasis under Fe Stress in Arabidopsis. J. Exp. Bot..

[111] Saini R., Singh Saini H., Dahiya A., Ritu Saini C. (2017). Iron Treatment Enhances the Levels of Reduced Glutathione, Oxidized Glutathione and Glutathione Reductase Activity in Rice (*Oryza sativa* L.). J. Pharmacogn. Phytochem..

[112] Zhang L., Li G., Wang M., Di D., Sun L., Kronzucker H.J., Shi W. (2018). Excess Iron Stress Reduces Root Tip Zone Growth through Nitric Oxide-Mediated Repression of Potassium Homeostasis in Arabidopsis. New Phytol..

[113] Ramírez L., Bartoli C.G., Lamattina L. (2013). Glutathione and Ascorbic Acid Protect Arabidopsis Plants against Detrimental Effects of Iron Deficiency. J. Exp. Bot..

[114] Rai S., Singh P.K., Mankotia S., Swain J., Satbhai S.B. (2021). Iron Homeostasis in Plants and Its Crosstalk with Copper, Zinc, and Manganese. Plant Stress.

[115] Zamioudis C., Korteland J., Van Pelt J.A., Hamersveld M., Dombrowski N., Bai Y., Hanson J., Van Verk M.C., Ling H., Schulze-Lefert P. (2015). Rhizobacterial Volatiles and Photosynthesis-related Signals Coordinate *MYB72* Expression in Arabidopsis Roots during Onset of Induced Systemic Resistance and Iron-deficiency Responses. Plant J..

[116] Romera F.J., García M.J., Lucena C., Martínez-Medina A., Aparicio M.A., Ramos J., Alcántara E., Angulo M., Pérez-Vicente R. (2019). Induced Systemic Resistance (ISR) and Fe Deficiency Responses in Dicot Plants. Front. Plant Sci..

[117] Pescador L., Fernandez I., Pozo M.J., Romero-Puertas M.C., Pieterse C.M.J., Martínez-Medina A. (2021). Nitric Oxide Signalling in Roots Is Required for MYB72-Dependent Systemic Resistance Induced by *Trichoderma* Volatile Compounds in Arabidopsis. J. Exp. Bot..

[118] Aparicio M.A., Lucena C., García M.J., Ruiz-Castilla F.J., Jiménez-Adrián P., López-Berges M.S., Prieto P., Alcántara E., Pérez-Vicente R., Ramos J. (2023). The Nonpathogenic Strain of *Fusarium oxysporum* FO12 Induces Fe Deficiency Responses in Cucumber (*Cucumis sativus* L.) Plants. Planta.

[119] Asgher M., Per T.S., Masood A., Fatma M., Freschi L., Corpas F.J., Khan N.A. (2017). Nitric Oxide Signaling and Its Crosstalk with Other Plant Growth Regulators in Plant Responses to Abiotic Stress. Environ. Sci. Pollut. Res..

[120] Oláh D., Feigl G., Molnár Á., Ördög A., Kolbert Z. (2020). Strigolactones Interact With Nitric Oxide in Regulating Root System Architecture of *Arabidopsis thaliana*. Front. Plant Sci..

[121] Corpas F.J., González-Gordo S., Rodríguez-Ruiz M., Muñoz-Vargas M.A., Palma J.M. (2022). Nitric Oxide and Hydrogen Sulfide Share Regulatory Functions in Higher Plant Events. Biocell.

[122] Kohli S.K., Khanna K., Bhardwaj R., Corpas F.J., Ahmad P. (2022). Nitric Oxide, Salicylic Acid and Oxidative Stress: Is It a Perfect Equilateral Triangle?. Plant Physiol. Biochem..

[123] Rasheed F., Mir I.R., Sehar Z., Fatma M., Gautam H., Khan S., Anjum N.A., Masood A., Sofo A., Khan N.A. (2022). Nitric Oxide and Salicylic Acid Regulate Glutathione and Ethylene Production to Enhance Heat Stress Acclimation in Wheat Involving Sulfur Assimilation. Plants.

[124] Tripathi D.K., Bhat J.A., Ahmad P., Allakhverdiev S.I. (2023). Polyamines and Nitric Oxide Crosstalk in Plant Development and Abiotic Stress Tolerance. Funct. Plant Biol..

[125] García M.J., Romera F.J., Stacey M.G., Stacey G., Villar E., Alcántara E., Pérez-Vicente R. (2013). Shoot to Root Communication Is Necessary to Control the Expression of Iron-Acquisition Genes in Strategy I Plants. Planta.

[126] Yang Y., Ou B., Zhang J., Si W., Gu H., Qin G., Qu L.-J. (2014). The Arabidopsis Mediator Subunit MED16 Regulates Iron Homeostasis by Associating with EIN3/EIL1 through Subunit MED25. Plant J..

[127] Sauter M., Moffatt B., Saechao M.C., Hell R., Wirtz M. (2013). Methionine Salvage and *S*-Adenosylmethionine: Essential Links between Sulfur, Ethylene and Polyamine Biosynthesis. Biochem. J..

[128] Dubois M., Van den Broeck L., Inzé D. (2018). The Pivotal Role of Ethylene in Plant Growth. Trends Plant Sci..

[129] Zhu X.F., Zhu C.Q., Wang C., Dong X.Y., Shen R.F. (2017). Nitric Oxide Acts Upstream of Ethylene in Cell Wall Phosphorus Reutilization in Phosphorus-Deficient Rice. J. Exp. Bot..

[130] Melo N.K.G., Bianchetti R.E., Lira B.S., Oliveira P.M.R., Zuccarelli R., Dias D.L.O., Demarco D., Peres L.E.P., Rossi M., Freschi L. (2016). Nitric Oxide, Ethylene, and Auxin Crosstalk Mediates Greening and Plastid Development in Deetiolating Tomato Seedlings. Plant Physiol..

[131] Hartman S., Liu Z., van Veen H., Vicente J., Reinen E., Martopawiro S., Zhang H., van Dongen N., Bosman F., Bassel G.W. (2019). Ethylene-Mediated Nitric Oxide Depletion Pre-Adapts Plants to Hypoxia Stress. Nat. Commun..

[132] Wünsche H., Baldwin I.T., Wu J. (2011). S-Nitrosoglutathione Reductase (GSNOR) Mediates the Biosynthesis of Jasmonic Acid and Ethylene Induced by Feeding of the Insect Herbivore *Manduca sexta* and Is Important for Jasmonate-Elicited Responses in *Nicotiana attenuata*. J. Exp. Bot..

[133] Rustérucci C., Espunya M.C., Díaz M., Chabannes M., Martínez M.C. (2007). S-Nitrosoglutathione Reductase Affords Protection against Pathogens in Arabidopsis, Both Locally and Systemically. Plant Physiol..

[134] Pommerrenig B., Feussner K., Zierer W., Rabinovych V., Klebl F., Feussner I., Sauera N. (2011). Phloem-Specific Expression of Yang Cycle Genes and Identification of Novel Yang Cycle Enzymes in Plantago and Arabidopsis. Plant Cell..

[135] Yang L., Ji J., Wang H., Harris-Shultz K.R., Abd Allah E.F., Luo Y., Guan Y., Hu X. (2016). Carbon Monoxide Interacts with Auxin and Nitric Oxide to Cope with Iron Deficiency in Arabidopsis. Front. Plant Sci..

[136] Sun H., Feng F., Liu J., Zhao Q. (2017). The Interaction between Auxin and Nitric Oxide Regulates Root Growth in Response to Iron Deficiency in Rice. Front. Plant Sci..

[137] Shi Y.F., Wang D.L., Wang C., Culler A.H., Kreiser M.A., Suresh J., Cohen J.D., Pan J., Baker B., Liu J.Z. (2015). Loss of GSNOR1 Function Leads to Compromised Auxin Signaling and Polar Auxin Transport. Mol. Plant.

[138] Frungillo L., Skelly M.J., Loake G.J., Spoel S.H., Salgado I. (2014). S-Nitrosothiols Regulate Nitric Oxide Production and Storage in Plants through the Nitrogen Assimilation Pathway. Nat. Commun..

